# Data on lithofacies, sedimentology and palaeontology of South Rifian Corridor sections (Morocco)

**DOI:** 10.1016/j.dib.2018.05.047

**Published:** 2018-05-15

**Authors:** W. Capella, N. Barhoun, R. Flecker, F.J. Hilgen, T. Kouwenhoven, L.C. Matenco, F.J. Sierro, M.A. Tulbure, M.Z. Yousfi, W. Krijgsman

**Affiliations:** a Department of Earth Sciences, Utrecht University, 3584CD Utrecht, the Netherlands; b Université Hassan II Mohammedia, Fac. Sci. Ben M'Sik, BP7955 Casablanca, Morocco; c BRIDGE, School of Geographical Sciences and Cabot Institute, University of Bristol, Bristol BS8 1SS, UK; d Department of Geology, University of Salamanca, 37008 Salamanca, Spain; e ONHYM, 10050 Rabat, Morocco

## Abstract

We provide lithological, sedimentological and micropalaeontological descriptions of 39 sections and boreholes crossing the upper Miocene deposits of the Rifian Corridor. These deposits represent the sedimentary remnants of the marine gateway that connected the Atlantic to the Mediterranean in the late Miocene. Results from these 39 sites were adopted to reconstruct the palaeogeographic evolution of the gateway presented in the associated research article (Capella et al., 2018) [1]. For each outcrop we present a synthesis of field observations, lithofacies, key sedimentological features, planktic and benthic assemblages.

## Specifications Table

**Table:** 

Subject area	*Field geology*
More specific subject area	*Sedimentology, micropaleontology, biostratigraphy, palaeoenvironment reconstruction*
Type of data	*Text file, figures (x5)*
How data was acquired	*Geological survey, identification and analysis of cross-stratification in sandstone, sampling for micropaleontology in hemipelagic sediments*
Data format	*Text description of lithofacies, planktic and benthic assemblages, key sedimentological features. Representative examples are illustrated in figures.*
Experimental factors	–
Experimental features	*Collection of data was oriented in such a way to detect (i) the onset marine transgression in foredeep and associated wedge-top basins, (ii) the stages of foreland basin subsidence, and (iii) the regressive stage of basin fill associated with the transition to continental deposits.*
Data source location	*Morocco (specific coordinates are provided for each site in WGS84)*
Data accessibility	*Data is in this article.*
Related research article	*Capella W, Barhoun N, Flecker R, Hilgen FJ, Kouwenhoven T, Matenco LC, Sierro FJ, Tulbure MA, Yousfi MZ, Krijgsman W. Palaeogeographic evolution of the late Miocene Rifian Corridor (Morocco): reconstructions from surface and subsurface data. Earth Science Reviews, in revision.*

## Value of the data

• We provide unpublished field datasets that can shed light on the palaeogeographic evolution of the Rifian Corridor: a late Miocene, Mediterranean–Atlantic gateway that progressively restricted causing the Messinian Salinity Crisis (MSC).
• Field research was carried out to identify lithofacies and key sedimentary structures; characterise planktic and benthic foraminiferal assemblages; the nature of these assemblages combined with sedimentary observations allow reconstructing age, palaeoenvironment of deposition, and basin evolution.
• Methods in the fields of high-resolution biostratigraphy were applied to define maximum and minimum age of the sediments and improve the age-model of the Rifian Corridor.
• This dataset provides a frame of reference for future geological surveys focusing on the upper Miocene in northern Morocco.

## 1 Data

**Fig. 1:**
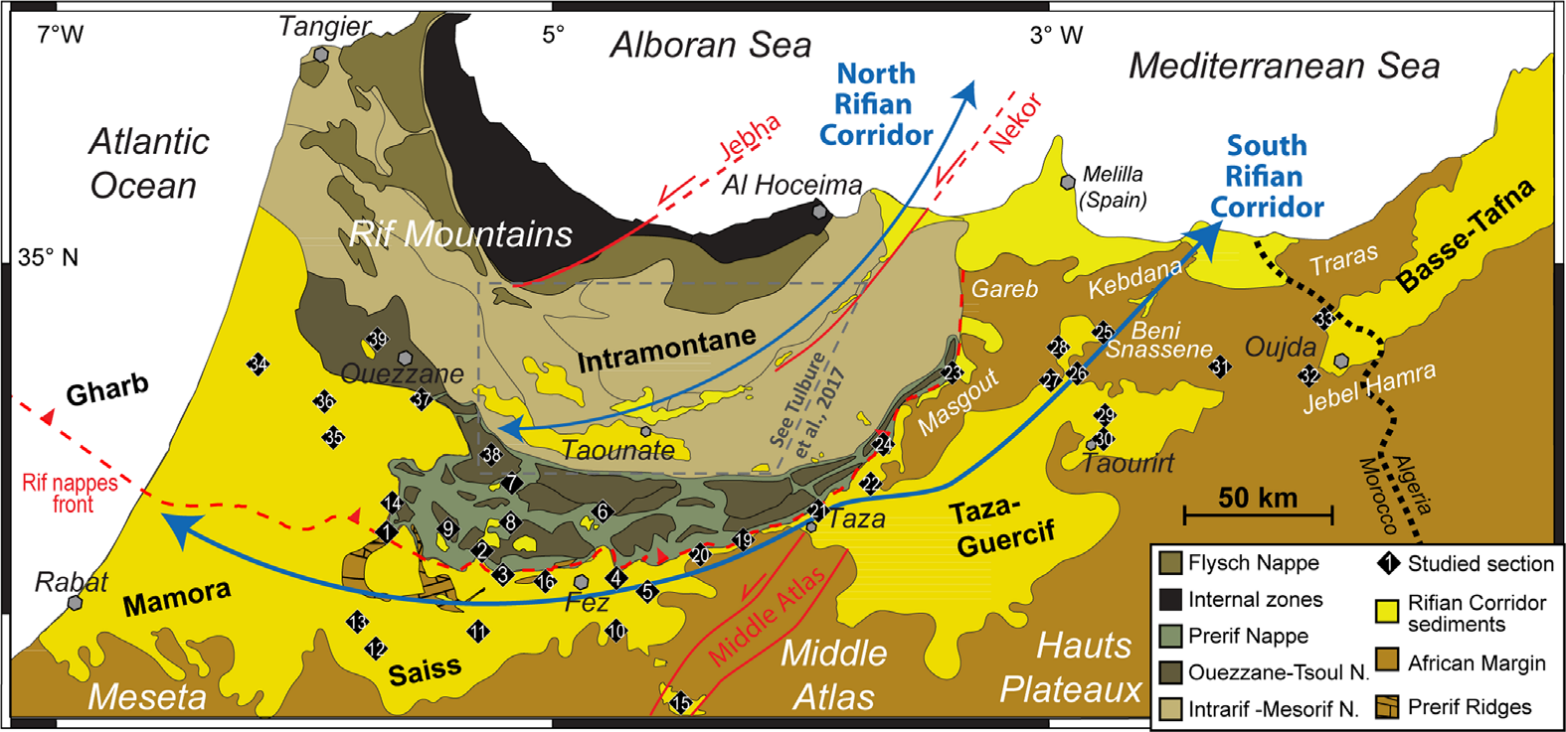
Simplified geological map of Northern Morocco with the tectonic units composing the Rif fold and thrust belt and the location of the studied sections. The Rifian Corridor sediments are upper Tortonian and lower Messinian deposits locally covered by Quaternary cover; they represent the approximate extension of the Late Miocene seaway. Tectonic units modified from [33], [34].

**Fig. 2:**
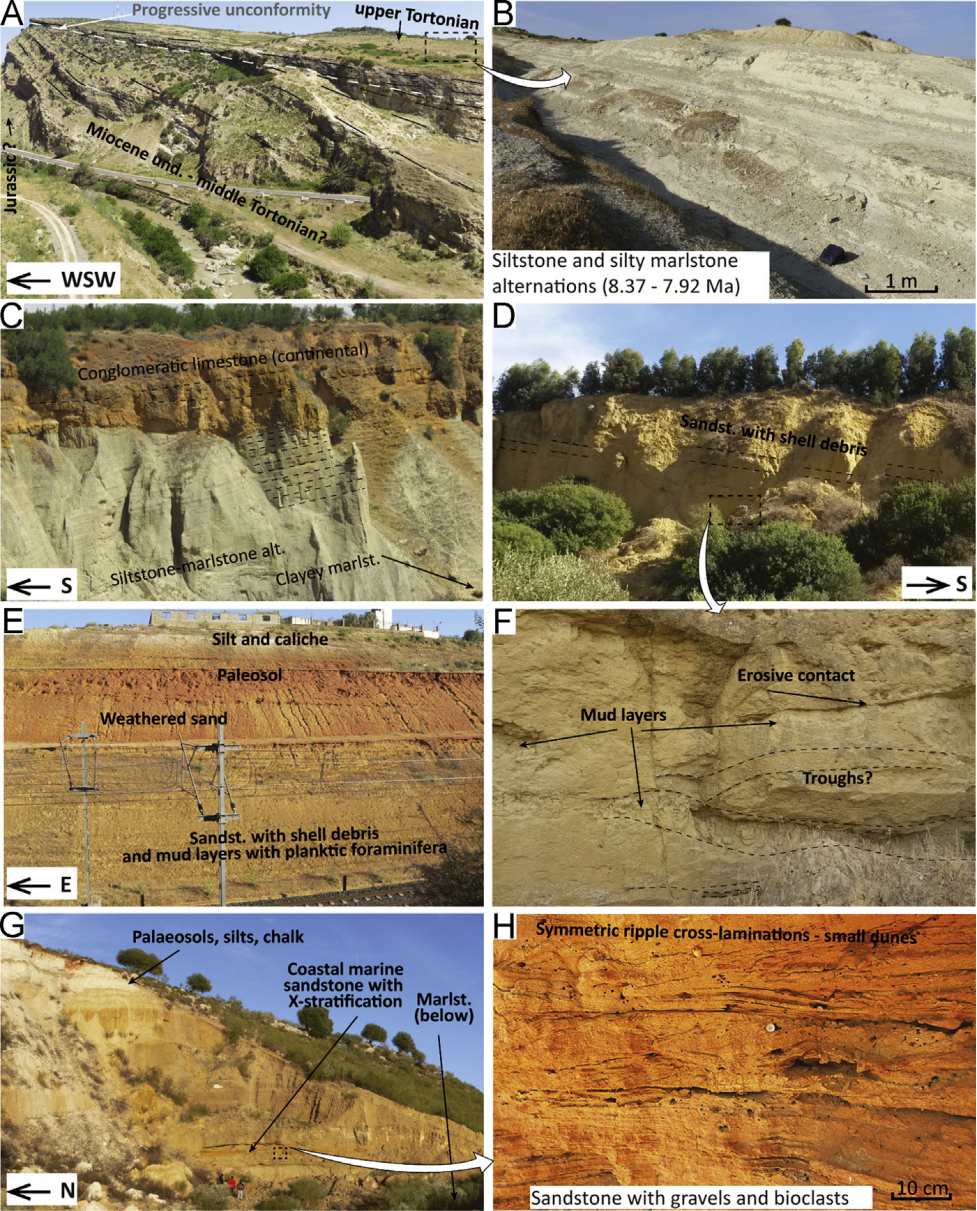

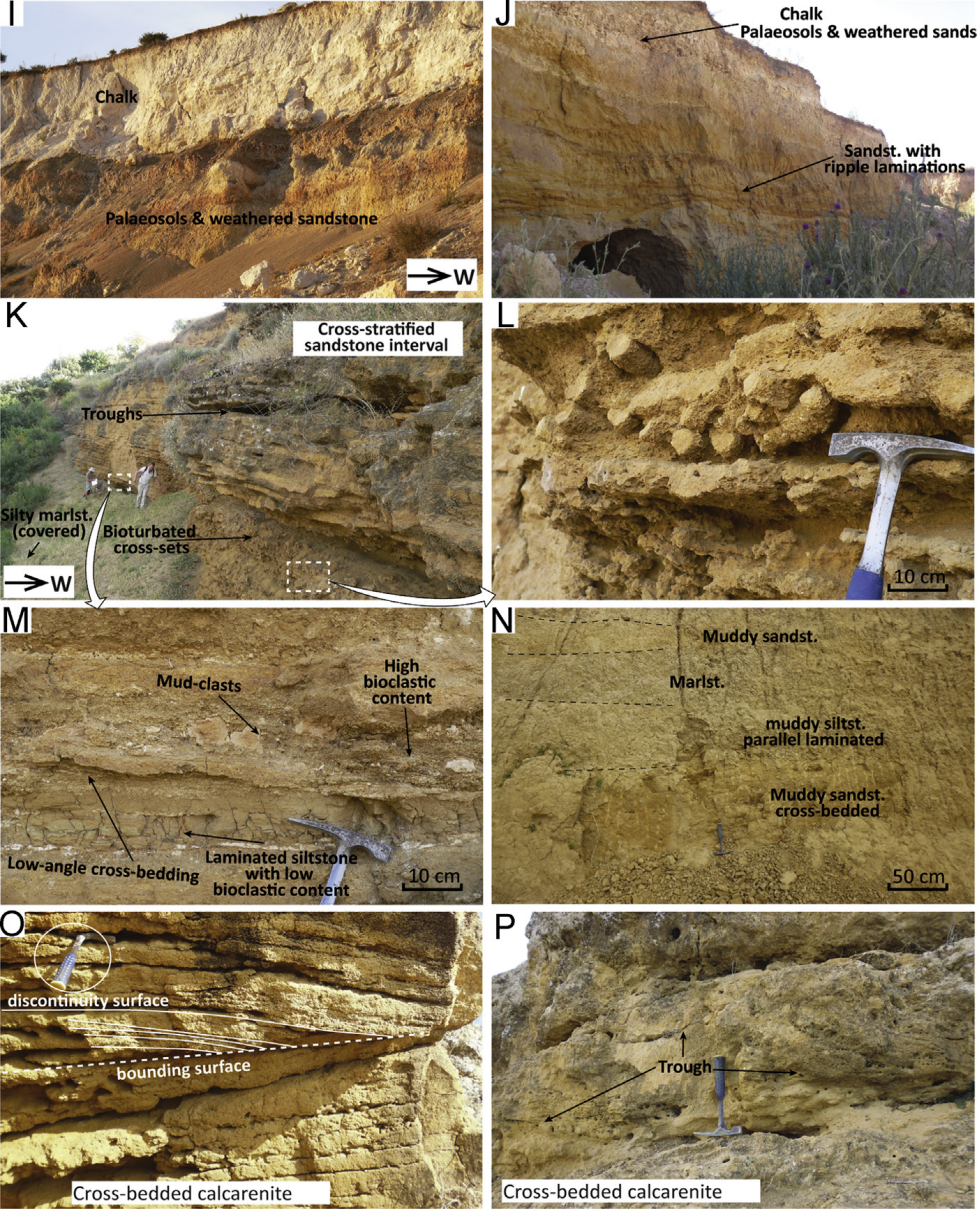
Field pictures of the sections in the Saiss Basin and Prerif Ridges area. (A) Progressive unconformity at the Bab Tisra section (Fig. 1, point 1) between older, undistinguished Miocene and upper Tortonian clastic limestone and silt-marlstone alternations (above and inset B). (C) Messinian silt-marlstones alternation truncated by conglomeratic limestone along the Madhouma section (Fig. 1, point 11). At an adjacent location (~ 2 km), the marine deposits grade upwards into a shallow-marine, yellow sandstone unit (D). (E) The same sandstone unit grading upward into continental deposits. (F) Erosional surfaces and cross-stratification in the sandstone unit shown in inset (D), in which palaeocurrents were measured (Fig. 1, point 11). The erosional surfaces dip-directions differ substantially from that of the average bedding attitude, which is to the southwest. (G) Outcrop view of the transition between coastal marine and continental deposits at the Ain Lorma section (Fig. 1, point 12). (H) is close up of the ripple cross-lamination in sandstone reflecting near-shore transport. (I) outcrop view at the Ain Lorma section of the contact between weathered, reddish sands with paleosols (*sables fauves* in [16]) and the overlying chalk and caliche-like deposits reflecting continental sedimentation. (J) The transition between coastal-marine sandstones and continental deposits is visible at multiple location of the Saiss Basin, this outcrop view is from ~ 5 km south of Meknes (section not on map). (K) Cross-stratified sandstone interval at the Bir Tam Tam section (Fig. 1, point 5). This interval is ~ 20 m thick and sandwiched between silty marlstones deposited at outer shelf depths. Palaeocurrent measurements indicate a west-directed flow. (L) Close-up of the bioturbated cross-sets. (M) Cross-strata at Bir Tam Tam showing mud-sand and bio-siliciclastic segregation; rip-up mud clasts are abundant in the bioclast-rich sets. (N) At Jebel Lemda, alternations of marlstone, muddy siltstone and cross-stratified muddy sandstone, contain slope/shelf edge-type benthic assemblages, and are therefore interpreted as contourites (Fig. 1, point 8). (O and P) Examples of cross-sets in Middle Miocene to middle Tortonian calcarenites, broadly reflecting SW-NE transport and attesting to a phase of wedge-top deposition above the orogenic wedge before 8.37 Ma.

**Fig. 3:**
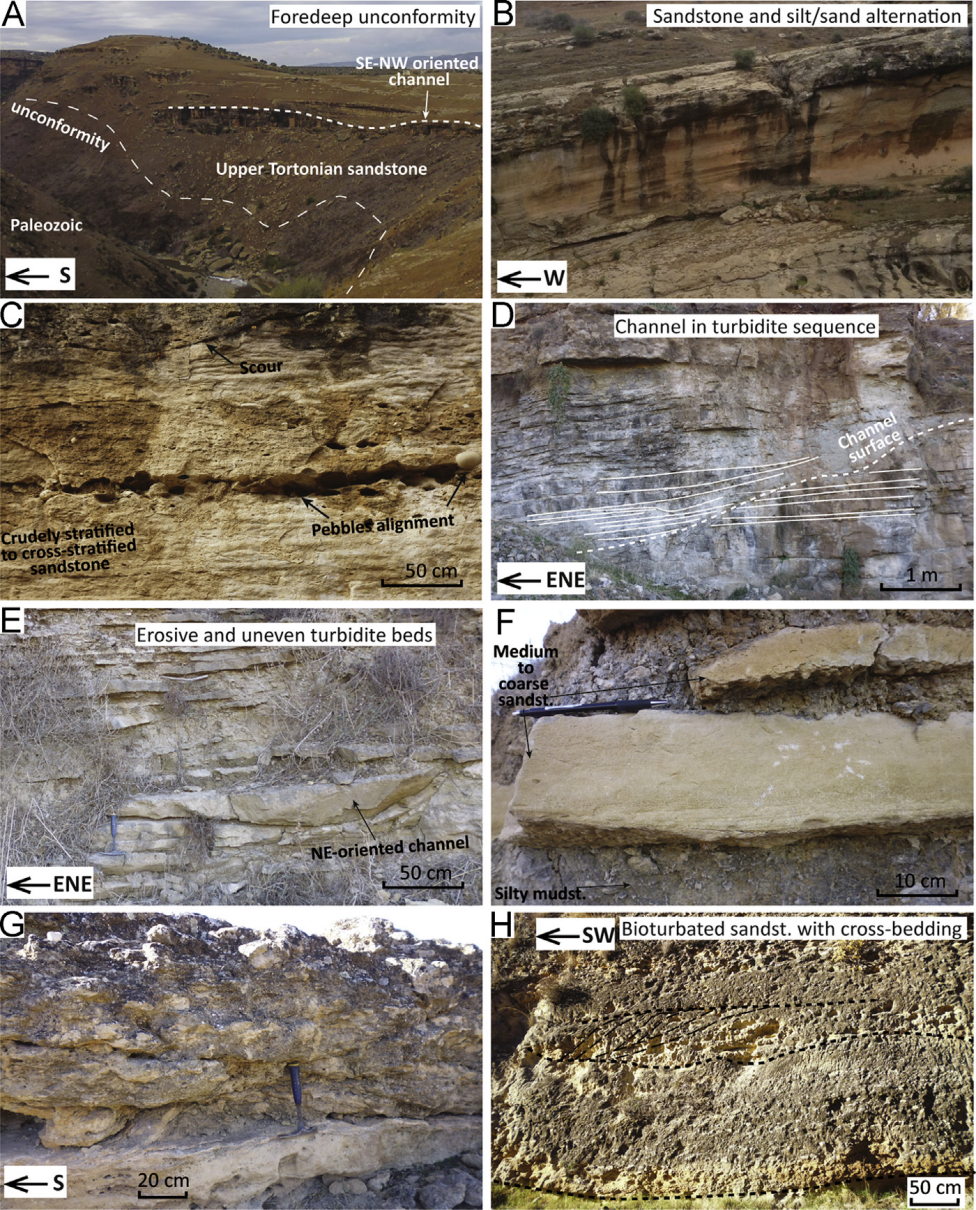

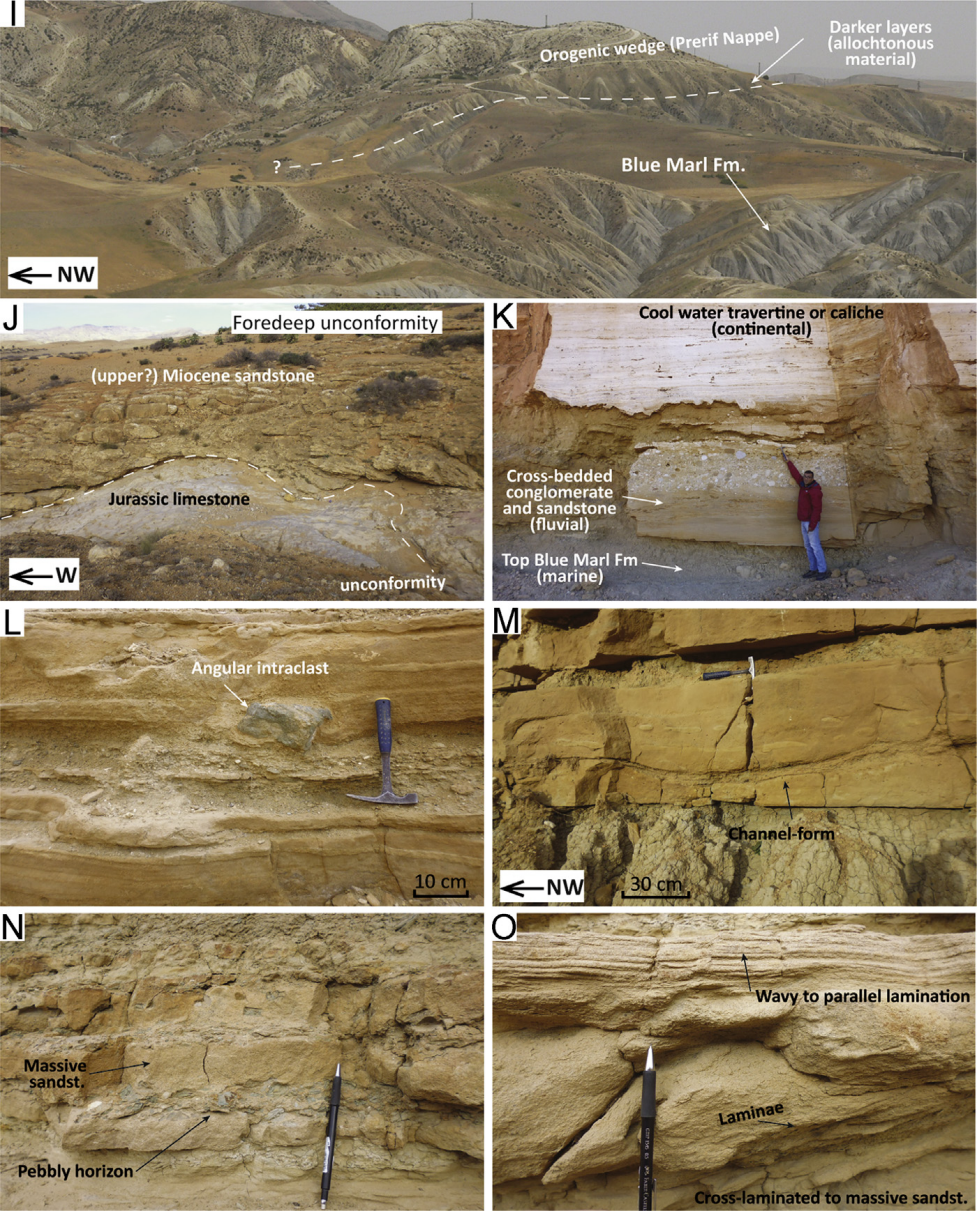
Field pictures of the sections in the Taza area. (A) At Col Touahar (Fig. 1, point 19, Palaeozoic schists of the Middle Atlas are incised by an erosional surface overlain by breccias and sandstones, grading upwards into pebbly sandstones and siltstones (B). (C) Close-up of the crudely- to cross-stratified sandstone with pebble alignments reflecting deposition close to a river fan-delta. (D, E) Channel-forms in turbidites (F) at Col Touahar. (G) Trough-cross bedding and (H) sigmoidal cross-bedding at Bouhlou (Fig. 1, point 20). (I) Panoramic view north of Taza (Fig. 1, point 21) showing the indistinct boundary between the Blue Marl Formation and the Prerif Nappe of the orogenic wedge. (J) Undifferentiated Miocene sandstones overlie unconformably the Jurassic limestones of the Masgout Massif at Ouled Bourima (Fig. 1, point 22). (K) Quarry exposure at Ouled Bourima showing the discontinuous transition from marine marlstones and continental deposits. (L–O) Close up on turbidites at the Ain Zohra section (Fig. 1, point 23).

**Fig. 4:**
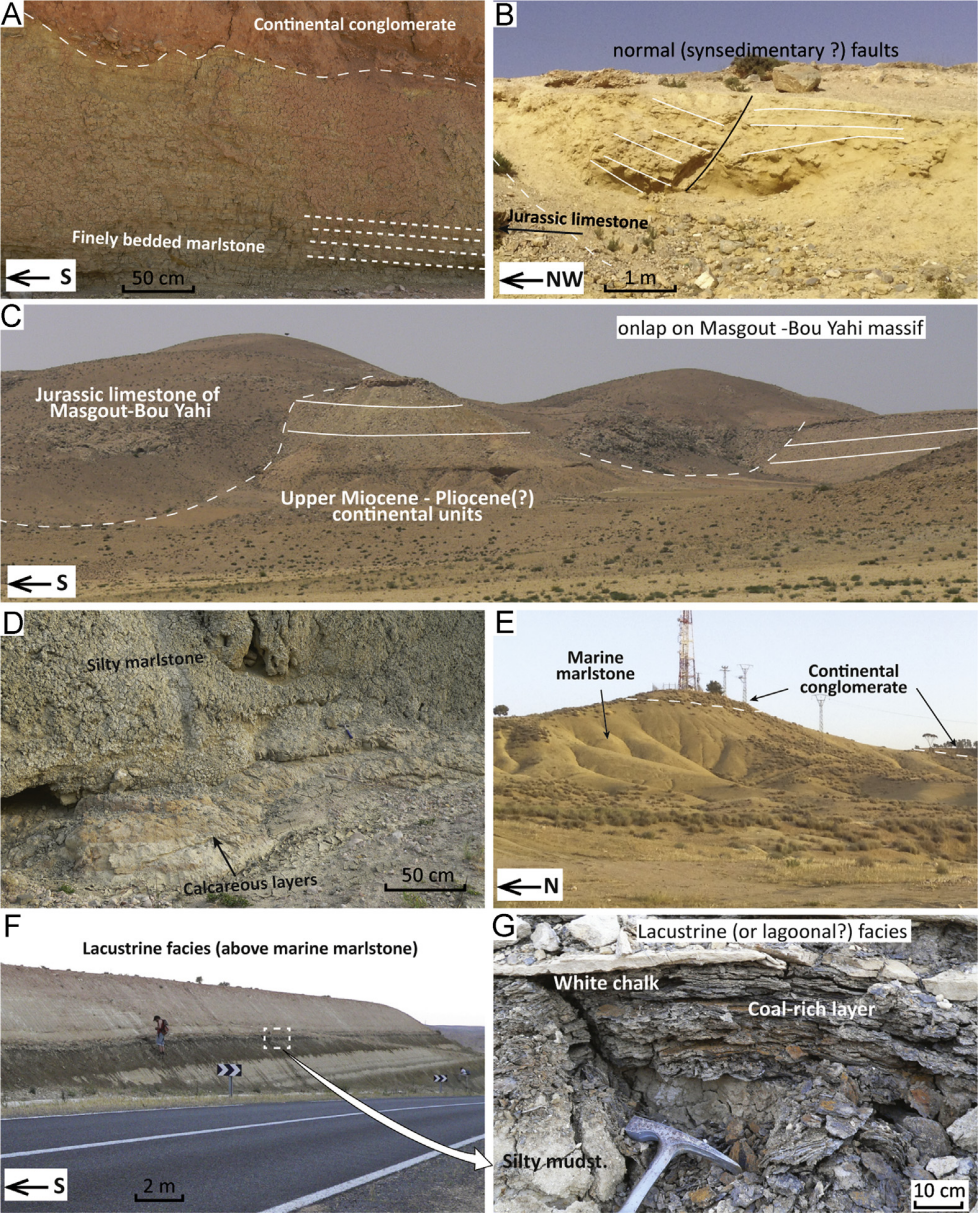
Field pictures of the sections in the Taourirt–Oujda area. (A) Middle Tortonian marlstones of the Hassi Berkane depocentre (Fig. 1, point 25). (B) Middle Tortonian deposits unconformably overlie Jurassic limestones by the shore of the Mohammed-V Dam (Fig. 1, point 26). Sandstone intervals display growth faults and are sandwiched between marlstones. (C) Onlap of the Upper Miocene or Pliocene continental units of the Hassi Berkane area on the Jurassic limestones of the Bou Yahi Massif (Fig. 1, point 28). (D) Alternation of silty marlstones and calcareous layers at the base of the Taourirt composite section (Fig. 1, point 29). (E) The top of section 29 (Fig. 1, point 29) truncated by Recent conglomerates. (F) The upper part (Fig. 1, point 30) of the Taourirt composite section records the transition from marine to continental deposition with (G) alternating levels of coal-rich layers, silty mudstone and white chalk, interpreted as lacustrine or lagoonal facies (Fig. 1, point 30).

**Fig. 5:**
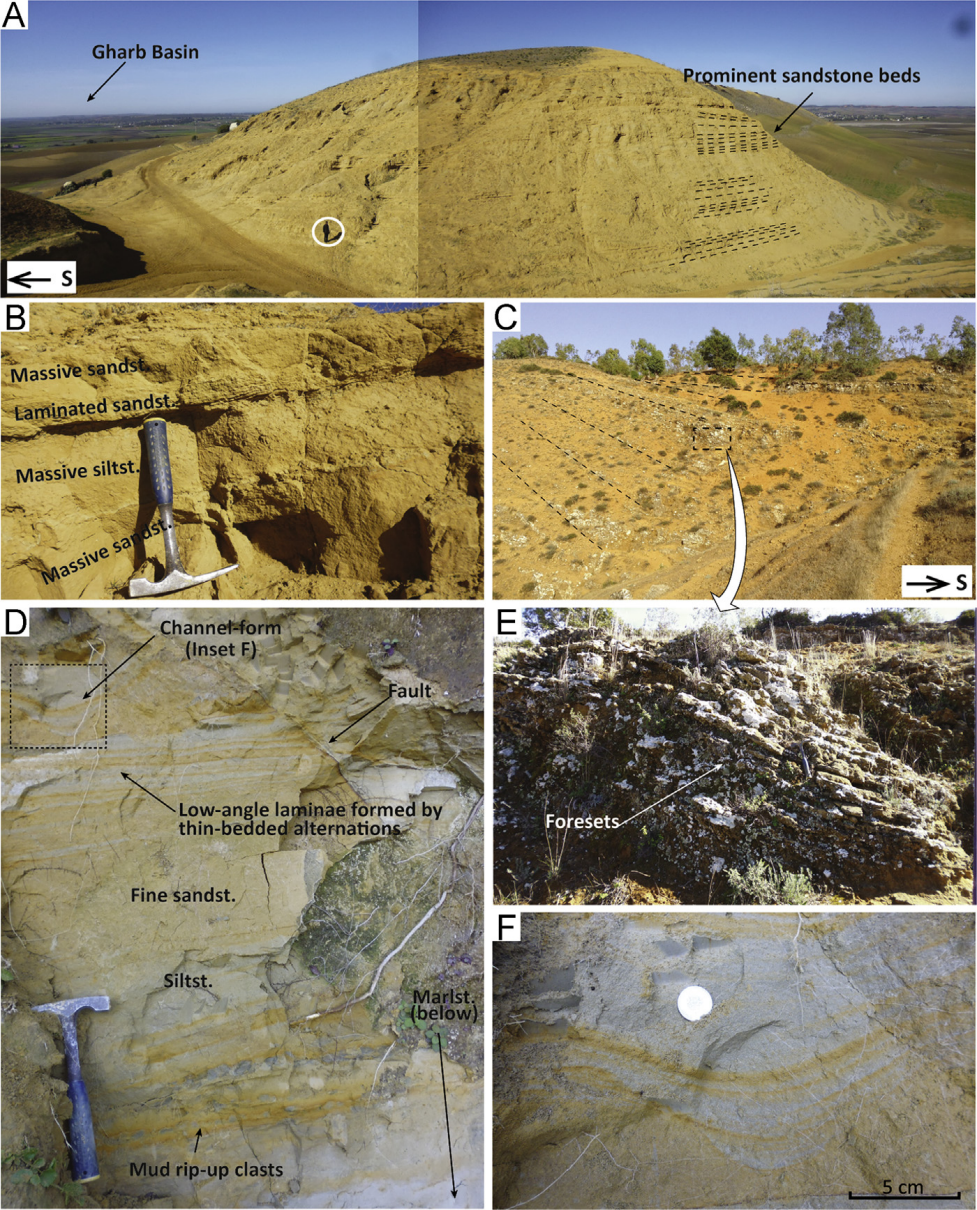

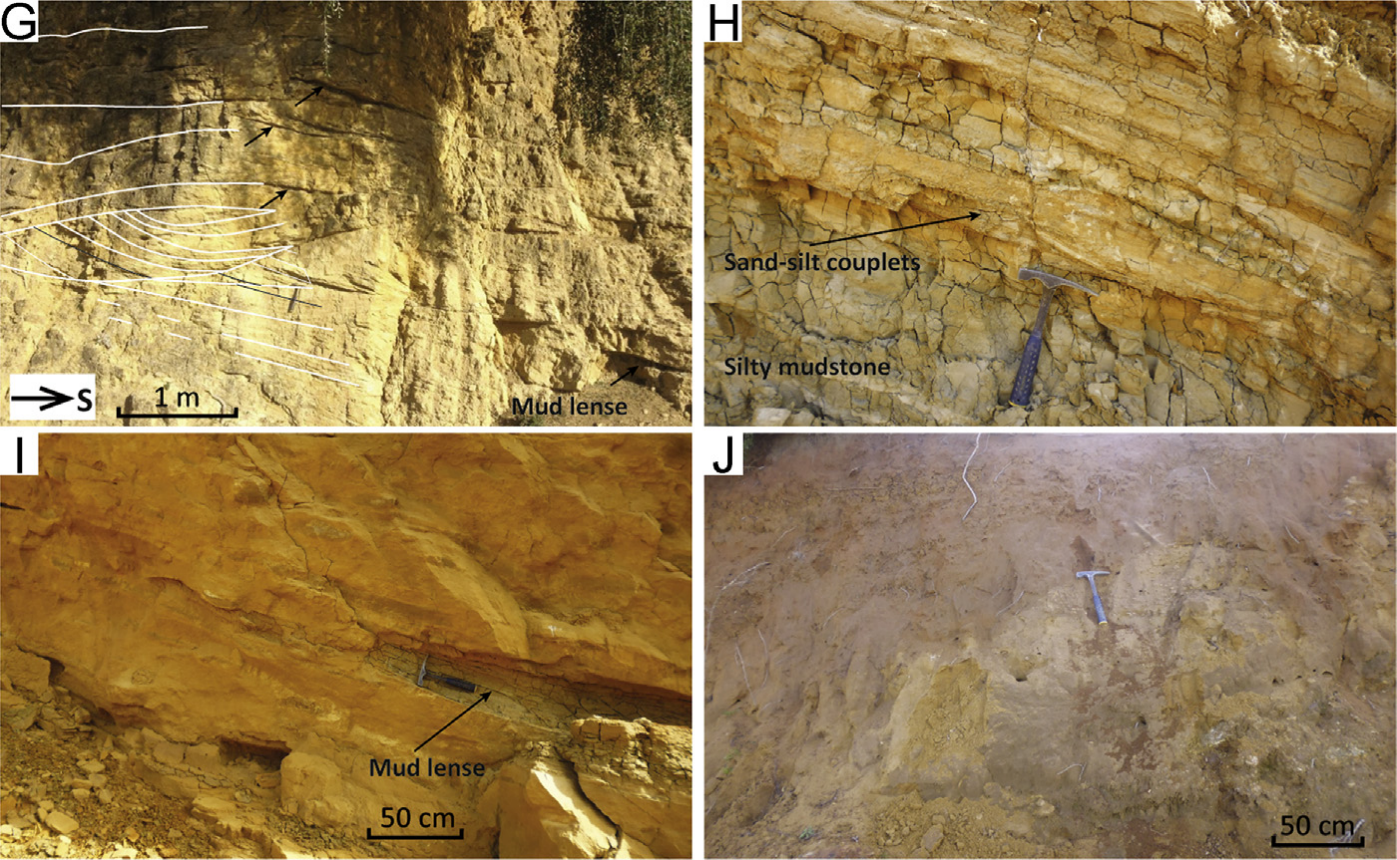
Field pictures of the sections in the Northern Gharb area. (A) Photomosaic showing an outcrop view of the Jebel Dhal (Fig. 1, point 34) turbidite sequence. (B) Details of the turbidites of Jebel Dhal. (C) Synkinematic wedge of coastal marine sandstones at El Sila (Fig. 1, point 37). (D) Close up of the siltstones-sandstones underlying the sandstones in (C) and showing alternating levels with channel-forms, low-angle cross-stratification, massive sandstone, siltstone and sandstone with rip-up clasts. (E) Close-up of the sandstones in (C) showing cross-strata. (F) Channel-forms are 10–15 cm in thickness and 5–10 cm in depth. (G) trough cross-bedding in sandstones at Moulay Abdelkrim (Fig. 1, point 38). Incisions are up to 1 m in depth. The black arrows indicate the base of different scours. In this position several mud-lenses are preserved and were sampled for biostratigraphy. (H) Close-up of the sedimentary structures observed at Mzefroun (Fig. 1, point 39). (I) Wedging-out of mudstone layer in a sandstone interval at Mzefroun. (J) Weathered and loose sands at the top of the Mzefroun section.

The dataset of this article provides detailed stratigraphic and lithological information of upper Miocene mainly-clastic sediments located in Northern Morocco. The analysis and characterisation of data allow reconstructing age and palaeoenvironment of deposition of the studied sections. This study spans 39 sites (Fig. 1), of which 6 boreholes and 33 outcrops, and supports the palaeogeographic reconstruction of the sedimentary basins of the Rifian Corridor presented in the associated research article [1]. In Section 3, we provide a concise summary of data analysis and characterisation, divided by areas according to the order followed in [1]. Outcrop and lithological descriptions are supported by field pictures (Fig. 2, Fig. 3, Fig. 4, Fig. 5). Analysis of planktic and benthic foraminiferal assemblages contained in the samples allows reconstructing age and palaeodepth of deposition. Bioevents of planktic foraminifera are numbered from 1 to 13; their absolute ages and relative references follow Table 1 of [1]. Lithological and sedimentological information was obtained on the site and integrated with previous works.

## 2 Experimental design, materials and methods

### 2.1 Biostratigraphy

Sampling for biostratigraphy was carried out in the finer-grained muds/marls, interbedded with the sandstones. A semi-quantitative analysis of the planktic foraminiferal marker species was carried out on the > 150 µm size fraction of the washed residue. Maximum–minimum ages of deposition are determined as result of the presence, relative abundance or absence of key planktic foraminifera species, namely, for the studied interval, *Neogloboquadrina acostaensis;* the keeled globorotaliids as *Menardella (Globorotalia) menardii* form 4 (here called *G. menardii* 4), form 5 (here called *G. menardii* 5)*,* and *Globorotalia miotumida.* In addition, also the sinistral or dextral coiling direction of *N. acostaensis* and *Globorotalia scitula* were used to determine age intervals, as well as the presence of the inflated form of *G. scitula* called *Globorotalia suterae* (see also events in Table 1 in [1]). This biochronology is based on an assemblage-based concept of the marker species whose first, last regular occurrence or coiling changes are tuned to the astronomical time-scale [2], [3]. For an extensive overview of taxonomic concept and marker species found in the upper Miocene in Northern Morocco, as well as comparisons with previously used concepts, see methods in [4].

### 2.2 Paleobathymetry estimates

The benthic foraminiferal assemblages from the washed fractions > 150 μm of selected samples were studied to estimate paleodepth and paleo-environment at time of deposition. Unlike the planktonic foraminifera that allow highly accurate biostratigraphy, many species of benthic foraminifera remained morphologically similar throughout the Middle-Late Miocene. Therefore, in each case we tested the planktic assemblage for possible reworking from older Miocene units. Planktonic-benthic (P/B) ratios were not considered reliable for depth estimates because most samples show signs of transport and some contain reworked specimens. Instead, semi-quantitative data of the benthic assemblages was used together with information on (differential) preservation, grain size sorting and reworking.

Depth-distributions of groups of benthic foraminifera known from literature were applied [5], [6], [7]. Although the slope profiles of the Rifian Corridor are likely to have been different from the continental margins on which these estimates are based, the distinction between shelf and slope type faunas is indicative of a shallower or deeper setting, respectively (see also the methodology part in [8], for an overview of the factors influencing benthic assemblages in the Rifian Corridor). In assemblages where both shelf- and slope-type species are present, and in absence of reworking, we considered that the shallow marine species (such as discorbids, *Ammonia*, *Elphidium* and *Rosalina* species [9]) were transported downslope.

### 2.3 Sedimentology

The measurement of palaeocurrent directions was carried out systematically in cross-bedded sets, in lithofacies such as cross-bedded sandstone and conglomerate. Because the data were acquired in deformed strata we corrected cross-bedding for tectonic tilt using the software *stereonet*. The data were then plotted using a non-linear frequency scale with *r*_1%_ = 1 cm [10]. Plotted data are shown in the maps of the 4 sub-areas (Figs. 3, 5–7 of the research article [1]).

## 3 Characterization and data analysis

### 3.1 Prerif Ridges area, Saiss Basin and associated outcrops (Figs. 3, 4 of [1])

#### 3.1.1 Bab Tisra

The Bab Tisra section is located a narrow canyon 5 km south of the town of Sidi Kacem (34.1815; − 5.7030). A panoramic view (Fig. 2A) shows a laterally extensive angular discontinuity in Miocene clastic units. The Miocene overlies unconformably the Jurassic limestone successions forming Mount Bou Draa (see Fig. 3 in [1]), which is part of the wider structure of the Prerif Ridges [11]. The intra–Miocene angular discontinuity showed in Fig. 2A marks the boundary between shallow marine, calcarenite deposits (M1 and M2) and a deepening–upward sequence (M3), which grades from basal conglomerates and packstones to marlstone (Fig. 2B). These overlying marlstone were sampled for biostratigraphy. For a description of M1 and M2, see [12], [13].

The discontinuity at the base of M3 is a progressive unconformity, with the oldest strata being more tilted than the youngest ones (Fig. 2A); the underlying units may therefore be part of the upper Tortonian sequence without major depositional hiatus occurring. M3 starts with partially cemented bioclastic sandstones with channels and reworked material from the sequence boundary below. This is overlain by a 2–3 m thick bioclastic packstone, rich in quartz, echinoderms, red algae and benthic foraminifera. The packstone grades into marly siltstone (Fig. 2B) containing planktonic foraminifera and some phosphatic nodules. Above the siltstone the sedimentation continues with marlstone deposits.

##### 3.1.1.1 Planktic and benthic foraminiferal assemblages

The residues are very rich in planktonic foraminifera and contain almost no detritus or transported material. All samples contain *Globorotalia menardii* 4. In the lower 10 m of the section (samples T1–18), sinistral and dextral forms of Neogloboquadrinids are common; in the upper 10 m (samples T18–24), Neogloboquadrinids are dominantly sinistral. *Globigerinoides extremus* is present throughout, although rare. In the Mediterranean, the FCO of *G. menardii* 4 occurs at 9.31 Ma [2]; neogloboquadrinids are dominantly dextral before 9.51 [14], [15], both sinistral and dextral between 9.51 and 7.92 Ma, and then dominantly and continuously sinistral after 7.92 Ma (event 9). The presence of *G. extremus* in the upper 10 m indicates that age of deposition post-dates the FO of this species (8.37 Ma; event 10), whereas the coexistence of sinistral and dextral forms of neogloboquadrinids could indicate that it pre-dates the onset of predominantly sinistral forms occurring between 8 and 7.92 Ma (event 9). Therefore, the data indicate that the white marlstones of Bab Tisra are deposited between 8.37 and 7.92 Ma.

#### 3.1.2 Ben Allou

Ben Allou section is located in the northern margin of the Saiss Basin (34.1017; − 5.2992). It is composed of alternations of marlstones and mainly clastic sandstones unconformably overlying the southern edge of the orogenic wedge. An extensive description of the section is given in [8] comprising its micropalaeontological content and sedimentological significance. The palaeocurrent pattern (Fig. 3 in [1]), indicates southwest–directed transport.

#### 3.1.3 Moulay Yacoub composite

The Moulay Yacoub section is located on the northern margin of the Saiss (34.0903; -5.1708) and in part overlies the frontal thrust of the Prerif Nappe, in part the foredeep lying on the Mesozoic limestones of the African margin. The section consists of mud-dominated deposits, mainly silty to clayey marlstones, with irregular intercalations of sandstones. These sandstone intercalations are on average 10–20 m thick and consist of turbidites [13], [16]. The direction of sandstone-transport in these turbidite units was analysed in [13], indicating SW- to SSW-directed flow (Fig. 3 in [1]). The composite section is ~ 800 m thick, but may be thicker, uncertainties due to limited exposures. The upper part of the section is characterised by the transition from marine marlstones to lacustrine facies.

To date this succession we used the samples of the Wernli collection stored at the Ministry of Geology in Rabat and additional samples taken in the uppermost part of the section. The section, roughly 800 m thick, is composed of the two following units: the lower unit, which is 300 m thick and corresponds to the so-called ‘*coupe de la nouvelle piste*’, and the uppermost unit, which is ~ 500 m thick and corresponds to the ‘*coupe de l’Oued Moulay Yacoub’* [16].

##### 3.1.3.1 Planktic and benthic foraminiferal assemblages

###### 3.1.3.1.1 Coupe de la nouvelle piste (Wernli's samples: BD52–58)

The micropalaeontological assemblage is characterised by the presence of predominantly sinistral *G. scitula*, predominantly sinistral *N. acostaensis, G. menardii* 4*, G. apertura, S. seminulina*. For this subsection, the age interval is between event 8 (7.92 Ma) and 5 (7.51 Ma).

###### 3.1.3.1.2 Coupe de l’Oued Moulay Yacoub (samples BQ 167–173, 175, 176, 179–181, 184–187)

The micropalaeontological assemblage is characterised by the following species: *G. extremus*, present throughout; *N. acostaensis*, present both in sinistral and dextral forms, but dominantly sinistral; *G. scitula* group, present with both sinistral and dextral forms, sinistral forms becoming dominant above BQ171. Distinct changes of assemblages are recorded as follows: between sample BQ167 and sample BQ168, the micropalaeontological assemblage contains abundant *G. menardii* 4*,* then its abundance gradually decreases and the form *G. menardii* 5 becomes abundant in sample BQ173. This interval between BQ168 and BQ173 probably contains events 4 and 5 (7.51 and 7.35 Ma).

Furthermore, *G. scitula* group becomes dominantly sinistral in sample BQ171. This could correspond to event 6 (7.58 Ma), suggesting that event 4 and 5 may occur above the interval between the samples BQ170 and BQ171.

The replacement of the *G. menardii* group by *G. miotumida* occurs in sample BQ175, reflecting event 2 (7.25 Ma). Microfauna become rarer in samples BQ179 and BQ185, and absent in samples BQ186–187.

Benthic foraminifera were analysed in 10 samples between BQ168 and BQ180. The benthic assemblages from the base of the interval are well represented and diversified and would indicate, broadly, upper-slope depths (300–500 m). Key indicators are *Sphaeroidina bulloides, Uvigerina semiornata, Uvigerina peregrina, Planulina ariminensis* and *Siphonina reticulata.* In the middle part (samples BQ173 and 175) a transition occurs to a poorly preserved low–diversity assemblage dominated by shallow–water forms (*Ammonia* and *Elphidium* spp.), followed by gradual restoration of an open marine environment approximately at the shelf break (BQ 184 and 181).

#### 3.1.4 East Fes composite

This composite section is located in the eastern part of the Saiss Basin, 10 km east of the town of Fes, and its different parts are named and located as follows. Sidi Harazem section (SH): 34.0250; − 4.8653. El Adergha section (EA): 34.0747; − 4.8534. Ain Kansera section (AK): 34.1246; − 4.8477. The three sub-sections show a wide variety of facies, namely basal transgressive conglomeratic limestones covered by basinal foredeep turbidites and marlstones (SH), upper-slope contourites (EA) and coastal marine sandstones (AK). Lithological and sedimentological description, age and palaeodepth determinations, and facies analysis are presented and discussed in [8]. In addition to the sets of samples described in [8], we checked some of the samples of the Wernli collection pertaining to the mud intervals that alternate with or overlie the basal limestone at Sidi Harazem. These samples were collected from cuttings of the Sidi Harazem core [16] and record the first event of marine deposition in the area during the Late Miocene. The basal limestone interfingers with intraformational conglomerates, gravels, bioclastic sandstones, sandy marlstones.

##### 3.1.4.1 Planktic and benthic foraminiferal assemblages

###### 3.1.4.1.1 Sidi Harazem core (samples BD 11,13, 20, 22, 23, 24)

The planktic foraminifera are in general rare and badly preserved, being pyritized and covered by crusts. However, it was possible to detect that the fauna is rich in warm water species such as *Orbulina* and *Globigerinoides* species. The analysed assemblages are characterised by the following planktic foraminifera: *G. scitula, N. acostaensis, S. seminulina, O. universa, G. obliquus, G. immaturus, G. apertura, G. bulloides, G. trilobus*. One specimen of *G. menardii* 4 was found in BD20.

These planktic species are consistent with the age range between event 7 and event 5 (7.80–7.51 Ma). The presence of *S. seminulina* in all samples indicates that the marine transgression occurred after 7.92 Ma (event 8) at this location.

#### 3.1.5 Bir Tam Tam

The Bir Tam Tam section (34.0278; − 4.6730) is located in the eastern end of the Saiss Basin, ~ 6 km northwest of the town of Bir Tam Tam. Up to 20 m thick sandstone intervals are sandwiched in silty marlstone (Fig. 2K). The sandstones are characterised by high bioturbation, high concentration of bioclasts and heavy minerals, mud rip-up clasts and cross-stratifications (Fig. 2L, M). Intervening mud of a brownish colour occurs in either thin horizons or lenses within the cross-sets. Cross-stratification is present in sets with both trough and planar bases; low-angle foresets are locally intercalated with mud (Fig. 2M). The measurement of palaeocurrent directions revealed west-directed sediment transport at this location.

##### 3.1.5.1 Planktic and benthic foraminiferal assemblages

The marlstones contain abundant planktic foraminifera, with estimated relative abundances of 80% planktic and 20% benthic foraminifera. The assemblages are characterised by abundant and dominantly sinistral *G. scitula*, including *G. suterae* type, and both *G. menardii* 4 and *G. menardii* 5. Dominantly sinistral *N. acostaensis* is also abundant. The coexistence of common *G. menardii* 5 and *G. menardii* 4 with the presence of abundant and sinistrally coiled *G. scitula* indicates that this section post-dates event 6 (7.58 Ma) or event 5 (7.51 Ma), and pre-dates event 4 (7.35 Ma).

Samples BT1–4 contain inner shelf species (e.g., *Ammonia, Elphidium* spp.) together with species commonly found on the deeper shelf and upper slope (e.g., *Siphonina reticulata, Cibicidoides pachyderma, Sphaeroidina bulloides, Uvigerina peregrina, Melonis barleeanum*). Palaeo-water depth of deposition of this sequence ranges between 150 and 300 m (physiographic domains of outer shelf/uppermost slope).

#### 3.1.6 Boudhilet

The Boudhilet section (34.2380; − 4.8654) is an isolated exposure of Miocene sediments overlying the Prerifian Nappe, 10 km north of the coastal-marine facies of the Ain Kansera unit (AK in log 4). This outcrop shows an alternation of brownish marlstones and 10–20 m thick yellow calcarenite intervals (Fig. 2o). The calcarenites contain abundant bioclasts, coarse grains lithified by carbonate cement, bioturbation. Cross-bedding is present in sets that are up to 60 cm in thickness (Fig. 2o). Direction of migration is measured based on the foreset dip-directions, resulting in a bi-polar palaeocurrent pattern, towards the NE and SE. Synsedimentary, normal faults affect the calcarenite units and are sealed by overlying marlstones.

##### 3.1.6.1 Planktic and benthic foraminiferal assemblages

The washed residues are characterised by abundant foraminifera and good sorting. Percentages of planktic foraminifera are between 50% and 70%. Marker species comprise *G. menardii* 4, *G. lenguaensis*, *N. acostaensis. Neogloboquadrina acostaensis* is very abundant and predominantly sinistral, but dextral forms are also present. This assemblage would indicate an age interval between event 12 (9.51 Ma) and event 10 (8.37 Ma); such an age is supported by the absence of key late Tortonian species, namely *S. seminulina* and *G. extremus*.

The assemblage contains outer shelf foraminifera (~ 15% *C. ungerianus*, *C. lobatulus*, some *N. fabum*), *U. peregrina*, and some (transported) shallow species as *Hanzawaia boueana, Ammonia* and *Elphidium* spp. The benthic foraminiferal assemblages could indicate deposition between depths of ~ 150 and 300 m.

#### 3.1.7 Karia ba Mohammed

The lithological units of this section crop out around the town of Karia ba Mohammed and compose an isolated exposure of Miocene sediments above the Prerif Nappe. Alternations of medium to coarse sandstones form a ~ 50 m thick sequence outcropping south of the town (34.3516; − 5.2157). The sandstones show cross-bedded sets with both planar and trough-shaped base (Fig. 2P); paleocurrents obtained from foreset dip-directions indicate bi-directional transport, with a predominant north–east directed component. Finer levels with good potential for preservation of hemipelagic fauna were not found at this location; therefore the section was dated analysing samples collected in mud layers alternated with bioclast-rich sandstones mapped as the same unit in [17], outcropping 2.5 km to the W–NW (34.3582; − 5.2443). It is unclear whether this unit corresponds to the same age, or belongs to part of the allochtonous material incorporated in the Prerif Nappe below.

##### 3.1.7.1 Planktic and benthic foraminiferal assemblages

The planktic assemblage contains *O. universa*, very abundant *G. immaturus, Globoquadrina dehiscens, G. lenguaensis*, indicating Middle Miocene age.

Benthic foraminifera are scarce. Some *C. bradyi* and *C. wuellerstorfi* were found together with N*. barleeanum* and *Gyroidina* spp.; absence of shallow shelf taxa indicates that the palaeo-depth was at least outer shelf/upper slope.

#### 3.1.8 Jebel Lemda

The Jebel Lemda section is one of the satellite exposures of Blue Marl Formation that overlies the Prerif Nappe to the north of Ben Allou and Moulay Yacoub. These exposures potentially hide relevant information for palaeogeographic reconstruction, as they may represent the link between the southern and northern strands of the Rifian Corridor. Jebel Lemda is located 16 km to the N–NW of Moulay Yacoub and 15 km NNE of Ben Allou. The sequence starts with blue marlstones (34.2309; − 5.2431) and increases upward both in sandy content and sandy intercalations. The estimated thickness of these marlstones is 100–200 m, and that of the overlying silty/sandy marlstones is 100 m. Sandstone beds are more common towards the top (34.2368; − 5.2206), where they occur in thicknesses of 1.5–2 m and are alternated with muddy silt and mud (Fig. 2N). The sandstones are very fine to fine in grain size, they are composed of bioclasts, quartz and heavy minerals. Bioturbation is rare but present. Some of the sandstone beds contain cross-stratification, indicating palaeo-flow predominantly towards the N–NW.

##### 3.1.8.1 Planktic and benthic foraminiferal assemblages

Planktic foraminiferal assemblages comprise predominantly sinistral *N. acostaensis*, predominantly sinistral *G. scitula, G. menardii* 4*, G. extremus, G. immaturus, G. apertura*. The abundance of *G. menardii* 4 varies throughout the section. Some *G. scitula* are of *G. suterae* type. This assemblage suggests that the Jebel Lemda section was deposited between event 7 (7.80 Ma) and event 5 (7.51 Ma). Samples collected in the lower marlstones (J1, 2) show high percentages of planktic foraminifera (~ 90–95%) and excellent preservation. Samples (J5, 6) collected in the marlstones between silty or sandy intercalations show lower percentages of planktic foraminifera, and very good sorting of both foraminifera and bioclasts, a feature typical of environments that are swept by permanent bottom-currents.

Benthic assemblages indicate an evolution from the lower part of upper bathyal (400–500 m) towards slope/shelf edge-type depths at the top. The basal marlstones contain assemblages characterised by up to 10% *C. kullenbergi*, *Uvigerina peregrina*, *U. semiornata* (~ 10–15%), *Siphonina reticulata* (~ 7–10%), *Hoeglundina elegans* (4%), and some *C. bradyi.* The marlstones intercalated with the sandstone beds at the top contain more transported material (including some reworked Cretaceous species). The assemblage is characterized by *C. ungerianus* (up to 10%)*, N. fabum, C. dutemplei*, and *Globocassidulina subglobosa.*

#### 3.1.9 Beni Ammar

This section is composed of scattered outcrops of muds and silts overlying the Prerif Nappe, in great part covered by recent agricultural fields (representative marlstones crop out at *x* = 34.1569; *y* = − 5.4296). At some location we also found scattered outcrops of silty white chalk, in general overlying conglomeratic intervals. Residues of the samples collected in these white chalk levels contained no microfauna after washing.

##### 3.1.9.1 Planktic and benthic foraminiferal assemblages

The micropalaeontological assemblage indicates a late Tortonian age (between event 8 at 7.92 and event 5 at 7.51 Ma) for samples BN1 and 2, which were collected in marlstone. Typical planktic foraminifera are *S. seminulina*, abundant *G. menardii 4*, very abundant sinistrally coiled *G. scitula* in sinistral forms, abundant sinistrally coiled *N. acostaensis*, and *G. immaturus*.

The planktic assemblage represents 90–95% of the total foraminifera. The few benthics are relatively well preserved, with large specimens. The benthic assemblages contain *Nonion barleeanum*, *Karreriella bradyi, C. kullenbergi* (~ 20%), *C. bradyi* (~ 10%), thus reflecting deposition at water depths corresponding to the lower part of the upper slope; with a probable depth range of 600–800 m.

#### 3.1.10 Jenanat section (after [18], [19])

The Jenanat section is located in the south–eastern part of the Saiss, 12 km north of the town of Sefrou (33.9290; − 4.7480); it was described and dated with modern bioschemes in previous works [19], and palaeoenvironmental reconstructions based on benthic foraminifera were performed [18]. The upper Tortonian succession at Jenanat unconformably overlies the Mesozoic limestones and dolomites of the Middle Atlas domain. The succession starts with calcarenites containing abundant *Heterostegina* spp. and echinoderms, grading upwards into marlstones with variable silt content. The top of the sequence is truncated by erosion.

##### 3.1.10.1 Planktic and benthic foraminiferal assemblages

Previous works [19] identified bioevents in the marlstones of this section, which include our events 5, 4 and 2). The section therefore is mostly late Tortonian in age and then intersects the Tortonian–Messinian boundary, marked by event 2 (7.25 Ma), that was found ~ 10–5 m below the upper contact of the marly unit. [19] show that the top samples contain both *G. miotumida* and *G. menardii* 5.

Palaeo-water depth of deposition throughout this section was reconstructed in [18]. The benthic assemblages vary throughout and suggest that the section records a deepening–upwards sequence, from inner shelf depth (~ 30–50 m) during deposition of the basal sandstone to upper bathyal (~ 300–400 m) during the deposition of the upper marlstones [18].

#### 3.1.11 Madhouma composite

The Madhouma section is located on the national road between Fes and Meknes, 5 km east of the village of Oued Jadida (lower unit: 33.9350; − 5.3200. Upper unit: 33.9130; 5.3000). This section straddles the transition from marine to continental deposition. The lower section (where samples M1–47 have been collected) is exposed in gullies and ravines of the Madhouma river valley. It starts with massive, blue and clayey marlstone, rich in pectinidae, echinoderms, and bivalves. The marlstones increase upward in silt content, forming more prominent silty/sandy layers at the top that coarsen upwards. These layers allow an estimation of the bedding attitude (Fig. 2C), which is 12° towards the southwest. In the western ravine of the Madhouma valley (Fig. 2C) the silty marlstones are truncated by a 10–20 m thick conglomeratic limestone. This unit also contains sandstones, gravels, oolithic grainstones, oncolithic concretions, and reworked material from the Middle-Atlas units (mostly limestone and chert). In some parts, this unit shows reddish sandstones coated by mud and carbonate. Some layers are exclusively composed by hardened carbonate cement that binds grains of gravel, sandstone, clay, and carbonate concretions of the charae-tube type.

In the eastern ravine of the Madhouma valley (Fig. 2D) the conglomeratic limestone is absent; yellow sandstone crops out above the clayey marlstones with the same bedding attitude of the silty marlstones opposite the ravine (Fig. 2C). The yellow sandstone interval (Fig. 2D) is ~ 30 m thick and consists of fine sand and silt alternations that thicken upwards. An upward increase has been observed in size and content of shell fragments (mainly oysters and bivalves), as well in the occurrence of scour-based beds. Fig. 2F shows an example of the channels and through-like erosional surfaces affecting the sandbeds. Lenses of sand with higher mud content occur throughout, usually below the troughs (Fig. 2F). Trough surfaces dip to the northeast and southeast, indicating an approximate palaeocurrent direction to the east.

The upper part of the Madhouma section crops out along a railway cut (Fig. 2E), about 3 km to the south of the Madhouma valley. The section starts with yellowish sandstones containing shells and shell debris (mainly oysters and bivalves); intervening mud layers are present throughout. These sandstones become increasingly mottled and weathered towards the top, grading into palaeosol horizons and hardened crusts. This gradual contact is overlain by white silts and carbonate cement that binds detrital grains and roots.

##### 3.1.11.1 Planktic and benthic foraminiferal assemblages

The planktic assemblage of both lower and upper section is characterised by abundant *G. miotumida*, predominantly sinistral *N. acostaensis*, *G. extremus*, and predominantly dextral *G. scitula*. The estimate age is early Messinian, between event 2 (7.25 Ma) and event 1 (6.35 Ma).

Both at the top of the lower section (Fig. 2C, D) and in the upper section (Fig. 2E) the benthic assemblage is dominated by shelfal species, such as *Nonion fabum*, and *Ammonia* and *Elphidium* spp. This assemblage indicates depths between 50 and 150 m. In the lower section, the relative abundance of planktic foraminifera decreases upwards and, in the mud-layers of the upper section, reaches the lowest values (ratios of 5%).

#### 3.1.12 Ain Lorma

The Ain Lorma section is located in the western part of the Saiss Basin, 20 km west of Meknes (33.8741; − 5.7534). Exposures along the valley of the El Kell River show the transition from marine marlstones to sand-dominated coastal facies and the typical continental units of the Saiss (Fig. 2G). The section starts with blue marlstones (estimated thickness is ~ 100 m) that locally crop out in small gullies and ravines. The marlstones increase in silt/sand content upwards. Above the marlstones are coastal marine sandstones. These sandstones grade upwards into continental units (Fig. 2G, I). The sandy unit is 30–50 m thick, and consists of irregular alternations of coarse sandstones with cross-stratification and ~ 10 cm thick indurated layers composed of finer and better sorted sands. The sands are poorly to moderately sorted, medium to coarse in grain size. Some cm-wide channels are filled with gravels and pebbles. The coarse sandstones show the following features (Fig. 2H): erosional channels, trough-cross bedding, parallel lamination, and ripple cross-lamination (often symmetric). The paleocurrents obtained from foresets and laminae dip-directions in the sandstones indicate NW- and SW-directed flows. Thickness of the sets decreases upwards. Some layers are heavily bioturbated, and the occurrence of bioturbated layers increases upwards towards the whitish silt at the top. The whitish unit (silts and chalk in Fig. 2G) is composed of carbonate and binds some roots and gravels.

##### 3.1.12.1 Planktic and benthic foraminiferal assemblages

The two lowermost samples of the sequence (samples A1, 2) were collected in clayey blue marlstone and show very well preserved microfauna and percent values of planktic foraminifera (relative to total planktic + benthic) around ~ 70–80%. This percent value decreases upward to 30–40% in the silty marlstones (samples A5, 6). The planktic assemblage is characterised by dominantly sinistral *N. acostaensis, Globigerinoides sacculifer, G. extremus,* abundant *Globoquadrina altispira*, abundant *G. miotumida. Globigerina bulloides* is abundant in some of the samples. This assemblage suggests that Ain Lorma was deposited between 7.25 (event 2) and 6.35 Ma (event 1).

Benthic foraminifera show a shoaling upwards trend at Ain Lorma, from an upper bathyal to a shelfal environment, consistent with the decreasing– upward percent values of planktic specimens in samples A1–6. Samples A1 and 2 are characterised by the following assemblage: *C. ungerianus* and *C. dutemplei* with *Nonion soldanii*, *Pullenia bulloides*, *Sphaeroidina bulloides*, some *U. semiornata* and *Spiroplectammina carinata*. This assemblage suggests shelf edge/upper slope, between ~ 200 and 400 m.

Samples A3 and 4 contain benthic foraminifera *C. dutemplei*, *C. ungerianus*, *N. soldanii*, *U. peregrina*. The assemblage indicates a slightly shallower assemblage, characterised by less *S. bulloides*, and more *textulariids* and *Astrononion stelligerum.*

Samples A5 and 6 show indications of transport, with good grain size sorting and benthic assemblage indicative of shelf depths. This assemblage is dominated by shallow species such as *Ammonia* spp. (> 10%)*; Elphidium* (> 5%) *N. fabum* with *C. dutemplei*, *C. ungerianus*. Some deeper species are also present (e.g., *C. italicus*), which would indicate either slightly deeper environments than shelf, or reworking from the Middle Miocene sedimentary rocks in the source area.

#### 3.1.13 Douar Zaouia

Douar Zaouia section is located ~ 10 km north of Ain Lorma section (33.9519; − 5.7661). It shows an alternation of sandstones and marlstones forming the upper part of the Blue Marl Formation between Meknes and the River Beth area. Some of the sandstone beds consist of fine sands and silts, other beds contain coarser sands, abundant bioclasts and cross-stratification. The small number of palaeocurrent directions measured in 10–50 cm thick cross-sets indicates west-directed transport. The section is truncated by fluvial conglomerates and gravels that show large channels and trough-cross bedding reflecting migration towards the NE.

##### 3.1.13.1 Planktic and benthic foraminiferal assemblages

The two samples taken in the marlstones of the Douar Zaouia section are characterised by abundant and predominantly sinistral *N. acostaensis*, *G. apertura*, *G. immaturus*, and rare *G. miotumida*. The assemblage contains forms that are reworked from older units (*Globorotalia mayeri, Globoquadrina dehiscens*), and is overall consistent with the assemblage of the Ain Lorma section. The Douar Zaouia section was deposited after event 2 (7.25 Ma) and before event 1 (6.35 Ma). The benthic foraminiferal assemblage is characterised by species such as *Elphidium* and *Ammonia* spp. indicating inner shelf depth for this section.

#### 3.1.14 Haricha

The Haricha section is located at the eastern edge of the Gharb Basin (34.2868; − 5.6450). It consists of alternations of sandstones and marlstones unconformably overlying the orogenic wedge. An extensive description of the section and micropalaeontological assemblage is given in [13,8]; palaeocurrent pattern is indicative of west-directed transport. See also results and facies associations in [8].

#### 3.1.15 Gulf of Skoura

The Gulf of Skoura extends ~ 30 km further south than Jenanat section and overlies unconformably the Middle Atlas Jurassic units. We rely on field observations and dating presented in [16], [20], [21]. The lithological log of this area shows a phase of continental (lagoonal or lacustrine) deposition at the base, overlain by a shallow marine sequence composed of silts and silty marlstones. What is relevant of this section is a unit of bioherms about ~ 50 m thick and containing *Porites*, formed in a relatively shallow and restricted southern embayment of the Rifian Corridor. The proposed age for the section is late Tortonian/early Messinian (*G. conomiozea* occurs in the marlstones overlying the bioherms); further analysis is required to constrain more precisely the age of this carbonate platform.

#### 3.1.16 Jebel Trhat

Jebel Trhat is located 5 km west of the town of Fes (34.0659; − 5.0374). Upper Tortonian bioclast-rich limestones and silts unconformably overly the Mesozoic limestone of Jebel Trhat. This section was studied by [16], analysing sets of samples from different flanks of the mountain. We could analyse one of these sets, which is stored at the Ministry of Geology in Rabat (samples PQ375–378). The samples are not taken in stratigraphic order; however, planktonic foraminifera are very well preserved and allow estimating an age interval between event 8 (7.92 Ma) and event 2 (7.25), possibly straddling the Tortonian–Messinian boundary.

##### 3.1.16.1 Planktic and benthic foraminiferal assemblages

PQ 378 and 377 contain *G. menardii* with both forms 4 and 5; form 4 is predominant. *O. universa, S. seminulina, G.bulloides* are abundant. N. acostaensis is predominantly sinistral and common. *G. immaturus, G. apertura* and *G. trilobus* are also common for these samples. *G. scitula* group is rare and predominantly sinistral. PQ 376 and 375 show abundant *G. miotumida*; *G. trilobus* and *G. extremus* are also abundant. In these samples *N. acostaensis* and *S. seminulina* are rare. *G. scitula* is present and predominantly sinistral.

### 3.2 Taza area (Fig. 5 in [1])

#### 3.2.1 Col Touahar

Col Touahar is roughly located at the point of divide between the sediments pertaining to the Saiss and the Taza-Guercif basins. Along the national road that descends from Col Touahar towards the west (34.1925; − 4.1866), Palaeozoic schists of the Middle Atlas domain are incised by an erosional surface (Fig. 3A) overlain by breccias and sandstones, grading upwards into pebbly sandstones and siltstones (Fig. 3B). Erosional channels and pinch-out of strata are locally visible (Fig. 3A). Overlying the first unit of breccia and sandstones are coarse-grained, poorly sorted, massive to stratified sandstones with abundant bioclasts, bioturbation, pebbles, and mud rip-up clasts (Fig. 3C). Massive clayey siltstones occur in lenses or layers. The lithofacies increases in silt and clay content towards the top (Fig. 3B), allowing sampling for more clayey material to date in micropalaeontological analysis (samples C2 and 3 collected at *x* = 34.1925; *y* = − 4.2037).

Some 3 km to the west and above some unexposed intervals the sequence continues with coarse sandstones passing upwards into a turbidite sequence (Fig. 3D–F) formed by sandstone and muddy silt alternations (sample C1 collected at *x* = 34.1885; *y* = − 4.2350). At outcrop scale, the turbidite sequence comprises characterising features such as lateral pinch-out of strata against a channel surface dipping 12° to the east (Fig. 3D), smaller channelised beds indicating palaeo-flow towards the NNE–NE (Fig. 3E) and crudely stratified single beds showing divisions of parallel to low-angle cross lamination (Fig. 3F). Lower contacts are moderately to deeply erosive and show abundant bioturbation, whereas upper contacts are quite distinct, wavy and sharp. Some cm-wide scours at the base of the some beds suggest a secondary direction of flow towards the NW.

The sandstone unit overlying the Paleozoic is characterised by alignments of pebbles (as in Fig. 3C) covered by laminated to cross-bedded sandstones. This superposition of facies is often observed at the mouth of river deltas in foreland settings [22] and reflects the partial bypassing of high-density turbulent flow (component of the hyperpycnal flow) above the basal dense flow. When the basal dense flow collapses for frictional freezing aligned layers of pebbles deposit, and as the turbulent flow bypasses the area cross-stratified to parallel-laminated sand accumulate. This scenario is also consistent with abundant rip-up clasts and scours present throughout the section (Fig. 3C).

The overlying turbidite sequence (Fig. 3D–F) may represent a more distal facies of the flood–dominated fan-delta system, namely sandstone with HCS (hummocky-cross stratification), commonly found basinwards of river mouths in foreland settings [22]. The sand-dominated deposition shows large northward conduits with erosive margins (Fig. 3D). The sand beds are parallel-laminated (Fig. 3F), channelised (Fig. 3E), and show sharp contacts. The wavy and sharp upper contacts of these graded sand beds indicate HCS, therefore wave-modified turbidites [23]. These facies may be the product of flood–generated sandstone lobes and represent a relatively proximal setting, where the hyperpycnal flows are still modulated by wave action [24], [25]. Furthermore, the contiguous sequence exposed at Bouhlou (see Section 2.2) also shows lithofacies consistent with delta-front generated deposits.

##### 3.2.1.1 Planktic and benthic foraminiferal assemblages

Three samples (C1–3) collected in the silty fraction of this section were poor in microfauna. Very few planktic foraminifera, abundant aggregates between specimen, fine sandstones and pyritized specimens hampered an accurate dating of the section. However it was possible to identify assemblages characterised by *G. bulloides*, *G. apertura*, *G. menardii* 4 and *G. immaturus*, which overall indicate a Tortonian age prior to event 5 (7.51 Ma). Given the regional transgression of Upper Miocene clastics on the Middle Atlas domain being well constrained between 8.37 and 7.92 Ma in the Saiss and Taza-Guercif Basins, these units are part of the late Tortonian biozone (8.37–7.25 Ma).

Benthic foraminifera are all hyaline and poorly preserved; the assemblages are dominated by epifaunal/epiphytic taxa (*Cibicides* spp.; *Elphidium* spp.; discorbids), suggesting shelf water-depths (100–200 m).

#### 3.2.2 Bouhlou

Next to the village of Bouhlou, along the railroad track, it is possible to analyse continuous outcrops of cross-stratified sandstones (34.1280; − 4.4061). These outcrops are mapped as the basal Upper Miocene unconformably overlying the Middle Atlas [17], part of the same unit of Col Touahar. The outcropping units at Bouhlou lack finer sediments with in situ micropalaeontological assemblages; therefore, biostratigraphic analysis was not carried out. However, given the good degree of lateral continuity between Col Touahar and Bouhlou, Bouhou is considered part of the same upper Tortonian transgressive unit.

The sands are medium to coarse grained; they contain larger clasts, cemented nodules, bioclasts and bioturbation. Cross-stratification in these sandstones occurs in sets with trough-shaped base (Fig. 3G). Sigmoidal and down-cutting surfaces (Fig. 3H) mark the contact between massive and cross-stratified sandstones. Palaeocurrent reconstructions from foreset dip directions show that coastal units were overall prograding towards the WNW at this location. Minor components to the south and north are possibly due to the local orientation of the troughs rather than transport towards other directions.

#### 3.2.3 Taza – scattered samples

We collected 12 samples in the available exposures of the Blue Marl Formation at several scattered locations to the north and northwest of the town of Taza. Here the blue marls crop out discontinuously in small gullies or along the banks of small brooks. A panoramic view shows the diffuse boundary between the blue marls and the Prerif Nappe of the orogenic wedge (Fig. 3I). Distinction between the two is often complicated by the fact that they form roughly the same badland landscape. Allochtonous material is usually marked by darker or whitish colours in the rocks, which contrasts with the blueish colour of the Blue Marl Formation (Fig. 3I).

##### 3.2.3.1 Planktic and benthic foraminiferal assemblages

The planktic foraminiferal assemblages of samples TZ1–12 are characterised by the following marker species: common *G. menardii* 4, predominantly sinistral *G. scitula*, predominantly sinistral *N. acostaensis*, *G. extremus* and, only locally, *S. seminulina. G. sacculifer* group is represented by *G. trilobus and G. immaturus*. The *G. apertura* group appears in high numbers. We therefore interpret these assemblages to be deposited between event 10 (8.37 Ma) and event 5 (7.51 Ma). Due to the absence of *G. seminulina* in some of the samples it is not possible to further restrict this interval using event 8 (7.92 Ma) as maximum age.

The benthic assemblages have mixed shelfal and shelf-edge species. Based on the common occurrence of *cibicidids* and *Uvigerina peregrina*, together with inner shelf species such as *Ammonia* spp. and *Nonion boueanum,* but in the absence of species convincingly indicating deeper (slope) environments we infer a palaeodepth of mid-outer shelf.

#### 3.2.4 Ouled Bourima

The Ouled Bourima section is located in the north–eastern part of the Taza-Guercif basin, 29 km north–east of the town of Taza. In this area a narrow band of Upper Miocene sediments extends northward from the Taza depocentre, wedged in between the Rif nappes to the west and the Masgout massif to the east. Miocene transgressive sandstones lie unconformably above the Jurassic limestone of Masgout (Fig. 3J). Moving ~ 2 km to the west, one quarry (34.3413; − 3.7320) shows the transition between marine marlstone and continental deposits (Fig. 3K). A clear erosional surface truncates the marine Blue Marl Formation; the sandy unit lying above is ~ 1 m thick, lacks shells or bioclasts, and contains foresets. These cross-sets are laterally continuous and indicate a south–westward direction of palaeo-flow. The sandy unit is truncated by a clast-supported conglomerate, which grades upward into clastic limestone formed by caliche-like carbonate concretions, consisting of hardened cement binding grains of gravel and pebbles. The clastic limestone unit grades upward into travertine-like facies, showing regular alternations of hard carbonate crusts and soft layers containing more terrigenous material.

##### 3.2.4.1 Planktic and benthic foraminiferal assemblages

Samples collected in the blue marlstones (samples O1 and 2) show assemblages containing predominantly sinistral *N. acostaensis*, *G. apertura, G. extremus,* predominantly sinistral *G. scitula* (some of which are G. suterae type), *G. menardii 4*, *O. universa, G. bulloides, G. immaturus*. These assemblages indicates an age between event 7 (7.80 Ma) and event 5 (7.51 Ma).

Benthic assemblages are relatively diverse, containing ~ 25% *cibicidids* (among others, 7% *C. kullenbergi*) and ~ 8% *Planulina ariminensis*. This assemblage, along with percentage of planktic foraminifera around 70%, indicates upper-slope depth (300–500 m).

#### 3.2.5 Ain Zohra

Upper Miocene facies wedged in between the orogenic units and the Masgout massif were analysed near the town of Ain Zohra (34.5892; − 3.6158). At this location, a ~ 30 m thick turbidite sequence unconformably overlies the Prerif Nappe. The sequence consists of crudely graded sand beds alternated with silts and silty muds. The sands are medium to coarse in grain size, poorly sorted, and locally contain larger grains, rip-up clasts or pebbles (Fig. 3L, N). The sand beds show flat to deeply erosive lower contacts, locally forming scours roughly SW-NE orientated (Fig. 3M). Some of these sand beds are amalgamated beds containing more than one depositional event; floating clasts seem to occur predominantly at the base of the beds (Fig. 3L, N). Divisions of massive, parallel- and cross-laminated sandstones are present (Fig. 3O). The laminated divisions are often wavy to slightly oblique (Fig. 3O). Palaeocurrent patterns reconstructed from cross-laminae suggest transport towards the NE–E, consistent with the main orientation of the scours (Fig. 3M).

The Ain Zohra turbidite sequence shows the superposition of fine-grained sediments (silty mud) and dam–thick sandstone lobes; these lobes have high sand-shale ratios (up to 8:1), channelised features and high numbers of superposed flow events visible in amalgamated beds up to 2 m thick (Fig. 3M). The beds are also rich in clasts and pebbles, often angular, which do not belong to this formation in lithology (Fig. 3L, N) but come from the Rif nappes (Cretaceous shale). Vertical superposition of floating clasts and massive sandstone (Fig. 3N) are usually the product of frictional freezing of gravelly dense flow and near–bed suspension, respectively [22]. Other beds show the vertical superposition of cross-laminated, crudely laminated and parallel laminated divisions (Fig. 3O) which is described as the deposit of a turbulent flow, causing suspension and sedimentation with traction processes along the bed [22].

##### 3.2.5.1 Planktic and benthic foraminiferal assemblages

The residues revealed abundant grains of sand, some evidence of transport and reworking, based on the good sorting of the material and Cretaceous species, respectively. Planktic foraminiferal assemblages contain *G. bulloides, G. apertura, G. extremus, G. immaturus*, sinistrally coiled *N. acostaensis*, *G. menardii 4*. These assemblages suggest an age between event 10 (8.37 Ma) and event 5 (7.51 Ma).

Benthic foraminiferal assemblages mostly containing *C. ungerianus, Gyroidina* spp. and some *P. ariminensis* reflect outer shelf to upper slope depth (200–300 m).

#### 3.2.6 Core AKL 101

The AKL-101 core (34.4575; − 3.6604) is located within the Prerif Nappe domain in plan view, but revealed a ~ 1000 m thick sequence of undifferentiated Neogene [26] overlying the Jurassic unit of the Middle Atlas. Neogene clastics are overlain by ~ 50 m of Prerif Nappe. The age of the uppermost ~ 300 m of the Neogene sediments was refined with modern bioschemes [27], suggesting an age of 7.58–7.51 Ma (corresponding to our events 6 and 5.

### 3.3 Taourirt–Oujda depocentres (Fig. 6 in [1])

#### 3.3.1 Hassi Berkane composite

The area of Hassi Berkane has very few exposures and no available information on its structure at depth [16]. We sampled this area because it could represent a narrow palaeo-strait that connected the Guercif depocentre to the Mediterranean Sea (Fig. 1). The highest topography in the area corresponds to the Beni Snassene (Fig. 1) and Bou Yahi Mesozoic Massifs; the latter being an eastern prolongation of the Masgout Massif. We analysed three separated outcrops south of the town of Hassi Berkane. The first outcrop (point 25; [1]) is located south of the town of Hassi Berkane (34.7942; − 2.8967) and shows ~ 20 m thick finely bedded marlstones gently tilted towards the north (Fig. 4A). The marlstones are truncated by an unconformity and covered by continental conglomerate.

The second outcrop corresponds to point 26 [1]. It is found near the Mohammed-V Dam (34.6664; − 2.9406) and shows an alternation of m-thick sandstones sandwiched in finer-grained sediments (Fig. 4B). Jurassic limestone towards the NW (Fig. 4B) is unconformably onlapped by this marly-sandy sequence. Some of the sandstone lobes show synsedimentary faulting capped by overlaying marlstones (Fig. 4B).

The third outcrop corresponds to point 27 (34.6860; − 2.9942). This unit consists of an alternation of indurated chalk and clayey siltstone containing typical fresh water microfauna of continental facies. We could not locate marine, upper Tortonian–Messinian sediments in this area; possibly they lie below the continental facies. Subhorizontal layers of the continental facies (Fig. 4C) terminate against the Jurassic limestones of Bou Yahi at point 28 [1]. Close observations at outcrop 27 revealed an alternation of indurated white chalk with gastropods and ostracods and blue to brown siltstone. The sequence is capped by m-thick continental conglomerates.

##### 3.3.1.1 Planktic and benthic foraminiferal assemblages

Samples HS 1–4 (outcrops 25 and 26) contain similar planktic foraminifera assemblages, all indicating middle Tortonian age, older than 8.37 Ma. Characteristic species are *N. acostaensis*, predominantly dextral with very few sinistral forms, *G. menardii* 4, which is present but rare, and *G. scitula*, mainly dextrally coiled but with some sinistral forms. These assemblages are characterised mainly by the absence of *G. extremus*, which assigns an age older than event 10 (8.37 Ma), and the dominantly dextral form of *N. acostaensis. Neogloboquadrina acostaensis* is predominantly dextral between event 13 (10.57 Ma) and 12 (9.51 Ma), then varies randomly between sinistrally and dextrally coiling until event 9 (8–7.92 Ma). The presence of predominantly dextral *N. acostaensis* may then reflect an interval between 10.57 and 8.37 Ma. Samples from outcrop 27 resulted in very small residues with rare microfossils. Ostracods are present with both valves preserved, suggesting lacustrine or brackish environments and very little transport. Both benthic and planktic foraminifera are rare but present, and species such as *G. bulloides, N. acostaensis*, and *G. apertura* were identified. However, these specimens have certainly been reworked from Neogene marine deposits and have no implication for the age and depth of this section. Whereas for outcrops 27–28 we can infer a continental environment, for outcrops 25–26 a diversified benthic assemblage and bathyal species such as *C. wuellerstorfi* are present, indicating open marine environment and palaeo-physiographic domains as deep as upper slope.

#### 3.3.2 Taourirt composite

The Taourirt composite section is compiled as follows [1]: point 29 (34.5495; − 2.7662) shows the basal part of the Blue Marl Formation unconformably overlying the Jurassic; point 30 displays the upper part of the blue marlstones and the transition to lacustrine facies (34.4565; − 3.8929). Point 29 shows, as its base, alternance of marlstones and more indurated, calcareous layers (Fig. 4D). The section passes upwards into ~ 50 m thick clayey marlstones and it is truncated by continental conglomerates of probable Quaternary age (Fig. 4E). At point 30, silty muds are poor in microfauna, and grade upwards into an alternation of white chalk, coal-rich layers and mud (Fig. 4F, G).

##### 3.3.2.1 Planktic and benthic foraminiferal assemblages

The washed residues of samples TA1–10 (collected at point 29) contain relatively common *G. menardii* 4 in the lowermost part of the section (TA1–4), whereas it is rare in the upper part (TA5–10). By contrast the group of *G. scitula* including the *G. suterae* is abundant. These assemblages suggest an age between event 7.80 and 7.35. Event 5 (7.51 Ma) is probably located between samples T4 and 5, whereas the overlying part (samples T5–10) was probably deposited between 7.51 and 7.35 because *G. menardii 4* was either rare or absent.

The washed residues of samples TA11 and 12 (collected at point 30) contain abundant ostracods and gastropods, indicating a brackish or fresh-water environment.

Benthic foraminifera at point 29 are dominated by *Lenticulinids*, *Uvigerinids*, *Cibicidids* and other outer shelf species and the planktic/benthic ratio oscillates between 60% and 70%. These assemblages would suggest shelfal depths.

#### 3.3.3 El Aioun core

The El Aioun core was analysed by Wernli [16] to trace the marine connection eastward from the Taourirt to the Oujda depocentres. Unconformably overlying the Jurassic limestones are ~ 200 m of sediments exclusively composed of continental lithofacies. The continental unit consists of massive conglomerate, lacustrine mud and intercalated gravel beds. By Wernli's opinion [16] part of these lacustrine lithologies correspond to the Pliocene lacustrine facies observed south of the Bou Yahi Massif.

#### 3.3.4 Beni Oulik core

This core is located 15 km to the west of the town of Oujda and consists of ~ 250 m of marlstone with terrigenous intercalations. This sequence was analysed by Wernli [16] and revealed the westernmost relict of upper Tortonian marine transgression in the Oujda depocentre.

##### 3.3.4.1 Planktic and benthic foraminiferal assemblages

A poorly preserved planktic foraminiferal assemblage hampered a precise dating of the core, which is assigned to undistinguished upper Tortonian–Messinian [16]. However, the same marine lithofacies of the Beni Oulik core were found at the base of the Angad core. The Angad core (see Section 3.3.5) revealed the presence of *G. suterae* and the absence of *G. conomiozea,* whose biozone is roughly equivalent to the modern *G. miotumida* [2], [28]*;* an assemblage that corresponds to a late Tortonian biozone (7.80–7.25 Ma). In addition, R. Wernli [16] highlighted a shallow marine benthic assemblage characterised by *Ammonia*, *Nonion* and *Lenticulina* spp. indicating inner to middle shelf depths (0–100 m), both at the base and at the top of the muddy sequence of the Beni Oulik core.

#### 3.3.5 Angad

The Angad core [16] shows a ~ 600 m thick marine marly sequence that coarsens and shallows upwards, then grades into silts and clays with gravel beds reflecting lagoonal or lacustrine environment.

##### 3.3.5.1 Planktic and benthic foraminiferal assemblages

The assemblages described for this site [16] showed the presence of *G. suterae*, of the *G. scitula* group [29], [30], above ~ 200 m from the base of the core. In older taxonomic concepts [16], *Globorotalia suterae* was thought to be present after the FO of *G. extremus* (our event 10) and before the FCO *G. conomiozea. Globorotalia conomiozea* [31] is, according to modern bioschemes, a form of *G. miotumida* [28], whose regular occurrence starts at 7.25 Ma [2], [3]. *G. conomiozea* is absent from the Angad core and, remarkably, from the entire Oujda depocentre [16].

Furthermore, *G. suterae* described by Wernli [16] is the same form of the *G. scitula* group that occurs synchronously around the Mediterranean at 7.80 Ma [32]. Due to this modern calibration of bioevents it is reasonable to date the Angad core between event 7 (7.80 Ma) and event 2 (7.25 Ma).

### 3.4 Northern Gharb area (Fig. 7 in [1])

#### 3.4.1 Jebel Dhal

The Jebel Dahl section is located in the northern Gharb Basin, 8 km NNW of the town of Souk el Arba (34.7523; − 6.0364). The section comprises sandstone and silty marlstone alternations forming a 150–200 m thick sequence. The sand beds are crudely graded; divisions of massive, cross-laminated and parallel-laminated sandstones are present (Fig. 5B). The lower contact of the sand beds is slightly erosive; some basal scour-and-fill structures suggest palaeocurrents towards the west. Palaeocurrent reconstructions were carried out from cross-laminae in the sand beds. The predominant direction of flow is approximately westward; a smaller number of observations indicate flow towards the north and east.

##### 3.4.1.1 Planktic and benthic foraminiferal assemblages

The washed residues were rich in microfauna showing very good preservation. Good grain size sorting in the residues hints at transport. The planktic foraminiferal assemblage is characterised by abundant *G. miotumida*, predominantly sinistral *N. acostaensis*, *G. extremus*, *G. apertura*, and predominantly dextral *G. scitula*. These assemblages indicate an early Messinian age, between event 2 (7.25 Ma) and event 1 (6.35 Ma).

Benthic foraminifera are characteristic of outer shelf to upper slope (100–300 m) with relatively abundant *Ammonia* spp. (~ 20 up to nearly 50%) in the assemblages probably due to transport. The assemblages contain *C. ungerianus*, *C. dutemplei* (up to ~ 20%), *Valvulineria bradyana* and *Nonion fabum. Uvigerina peregrina* is present up to ~ 7%.

#### 3.4.2 NRT-2 core

Biostratigraphic analysis in the NRT-2 core was conducted by Barhoun [27]. The core showed ~ 1300 m of mostly clayey marlstones with some sandy intervals; the marlstones contain abundant *G. miotumida*, dominantly sinistral *N. acostaensis,* and dominantly dextral *G. scitula*, indicating an early Messinian age between event 2 (7.25 Ma) and 1 (6.35 Ma).

#### 3.4.3 Jebel Bibane

The Jebel Bibane section is located 8 km west of the town of Souk el Arba (34.6736; − 5.9191). The section shows a ~ 80 m thick sandstone interval encased in blue marlstones with varying silt content. At the base of the marlstone is a ~ 30 m thick calcarenite unit attributed to the upper Tortonian [13]. The sandstone interval has many clayey intercalations and lenses which allow sampling for microfauna. The sand beds are turbidites showing divisions of cross-lamination and parallel lamination; slumped levels are common throughout the section. Feeding turbidity currents broadly directed south to south–west were derived from palaeoflow indicators [13].

##### 3.4.3.1 Planktic and benthic foraminiferal assemblages

The residues of the samples collected in this section showed planktic and benthic assemblages analogous to those of the Jebel Dhal section, namely reflecting early Messinian (7.25–6.35 Ma) and outer shelf to shelf edge (100–300 m).

#### 3.4.4 El Sila

The El Sila section is located at the north–eastern margin of the Had Kourt depocentre (34.6721; − 5.5259) and consists of a thin sequence of marine, marly deposits capped by coastal sandstones, the latter mapped as Pliocene in the regional geological maps [17]. The marine marlstones contain shell fragments of bivalves, oysters, and gastropods, and grade upwards into alternations of clayey silts and very fine sands that show horizons of rip-up clasts, small channels, cross-lamination, and wavy lamination (Fig. 5D, F). These alternations increase upwards in sand content until the section is composed exclusively by sandstone (Fig. 5C). Sandstones become reddish and more weathered towards the top (Fig. 5E). The sandstones are crudely stratified or cross-stratified, with cm-thick sets. It is possible to observe m-thick sets of through-cross bedding in freshly cut exposures. The sands contain bioclasts and are coarse in grain size. Paleocurrent reconstructions from cross-laminae in the silt and larger foresets in the sandstones indicate NNE and SSW direction of transport. Layers become decreasingly tilted towards the top (Fig. 5C).

##### 3.4.4.1 Planktic and benthic foraminiferal assemblages

The washed residues are very large, containing great amount of bioclasts. Planktic foraminifera are abundant but seem mostly reworked, with Eocene, Middle Miocene and Tortonian species. The maximum age is Tortonian, but the sediment could be younger. Poorly preserved and scarce benthic assemblage containing *C. ungerianus*, *Pullenia bulloides*, *Oridorsalis* spp., *Ammonia* and a few *Elphidium* spp., indicate shelfal palaeo-water depths.

#### 3.4.5 Moulay Abdelkrim

At this location (34.4832; − 5.2446), unconformably overlying some Cretaceous units of the orogenic wedge are sandstones with abundant trough cross-bedding and mud lenses (Fig. 5G). The troughs are 60–80 cm high and 2 m wide. Mud lenses are preserved either at the top of a trough fill or between sigmoidal packages of foresets (Fig. 5G). Palaeocurrent reconstructions based on foreset dip-directions show east-directed transport. Above 4–5 m from the base, the trough-shaped surfaces are replaced by planar bedding.

##### 3.4.5.1 Planktic and benthic foraminiferal assemblages

The washed residues show a mix of bioclasts and planktic foraminifera. The planktic foraminifera are from different ages (e.g., Cretaceous, Eocene, Middle and Late Miocene). We could find some sinistrally and dextrally coiled *N. acostaensis*, and *G. menardii* 4; the specimens of *G. menardii* 4 are well preserved indicating that the in situ species are probably of Tortonian age. However, it is not possible to specify middle or late Tortonian from the microfauna.

Benthic foraminiferal assemblages are characterized by *C. dutemplei*, ~ 20% *C. ungerianus, C. lobatulus. Elphidium* and *Ammonia* spp are present and in part badly preserved. The estimated water depth is mid-outer shelf, but reworking of late Miocene precludes a more precise depth estimate due to uncertainty about which species are in-situ.

#### 3.4.6 Mzrefroun (composite)

This section is located 15 km east of the town of Ouezzane (34.8371; − 5.7453). The sequence starts with coarse bioclastic sandstones with cross-stratification, then grades upwards into mudstone in which ~ 10 m thick sandstone lobes are intercalated (Fig. 5I). These sandstone lobes include cross-laminated silt–sand couplets and massive to laminated mud lenses (Fig. 5H, I). The sands are fine to medium and moderately sorted, with bioclasts and coarser granules. The sequence increases in silt–sand content towards the top; the sands become more reddish in colour and medium to coarse in grain-size. Mud layers or lenses with organic material occur within heavily weathered and mottled sandstone (Fig. 5J). Gravel beds occur near the top. Cross stratification is present in sets of planar cross-bedding in the coarse sandstone at the top and the bottom of the section; cross-lamination in the sand–silt couplets intercalated with mudstone (Fig. 5H). Palaeocurrent directions based on cross-laminae in sandstone-lobes and cross-stratification in bioclastic sandstones can be indicative of the following processes that may change through time: tidal currents; sand bars progradation; long-shore wind-driven currents. The sandstone-lobes with mud-lenses (Fig. 5I) may reflect fan-delta sandstones and prodelta mudstones.

##### 3.4.6.1 Planktic and benthic foraminiferal assemblages

All the particles in the residues seem very well sorted due to transport. Probably 99% of the foraminifera are reworked from Cretaceous, Eocene, and Middle to Late Miocene units. We could identify some sinistral *N. acostaensis* suggesting that the maximum age is Tortonian. Benthic foraminiferal assemblages are dominated by relatively well-preserved *Ammonia* spp. together with *Elphidium* spp. and few other taxa living in (shallow) shelf environments. Together with plant remains this indicates a shallow to coastal marine environment (max. 100 m), probably with deviating salinity.
