# Supplementary material for: Data on lithofacies, sedimentology and palaeontology of South Rifian Corridor sections (Morocco)
Source: Data Brief. 2018 May 15;19:712–36. doi: 10.1016/j.dib.2018.05.047 (PMC6120058; doi:10.1016/j.dib.2018.05.047)

**Author declaration – Conflict of Interest Form**

We wish to confirm that there are no known conflicts of interest associated with this publication and there has been no significant financial support for this work that could have
influenced its outcome.

We confirm that we have given due consideration to the protection of intellectual property
associated with this work and that there are no impediments to publication, including the
timing of publication.

Yours sincerely,

Dr. Walter Capella (on behalf of all co-authors)


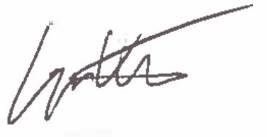

Supplement: Supplementary file 1 — Supplementary material [file mmc1.docx]
